# A first report on the efficacy of a single intra-articular administration of blood cell secretome, triamcinolone acetonide, and the combination of both in dogs with osteoarthritis

**DOI:** 10.1186/s12917-022-03413-2

**Published:** 2022-08-13

**Authors:** J. C. Alves, A. Santos, P. Jorge, L. Miguel Carreira

**Affiliations:** 1 Divisão de Medicina Veterinária, Guarda Nacional Republicana (GNR), Rua Presidente Arriaga, 9, 1200-771 Lisbon, Portugal; 2 Environment and Development, MED – Mediterranean Institute for Agriculture, Instituto de Investigação E Formação Avançada, Universidade de Évora, Pólo da Mitra, Ap. 94, 7006-554 Évora, Portugal; 3 Faculty of Veterinary Medicine, University of Lisbon (FMV/ULisboa), Lisbon, Portugal; 4 Interdisciplinary Centre for Research in Animal Health (CIISA), University of Lisbon, (FMV/ULisboa), Lisbon, Portugal; 5 Anjos of Assis Veterinary Medicine Centre (CMVAA), Barreiro, Portugal

**Keywords:** Blood cell secretome, Triamcinolone, Dog, Osteoarthritis, Pain, Hip

## Abstract

**Background:**

Osteoarthritis represents a significant welfare problem for many dogs, with limited therapeutic options other than palliative pain control. To evaluate the effect of the intra-articular administration of blood cell secretome and triamcinolone, 15 dogs with bilateral hip osteoarthritis were randomly assigned to a blood cell secretome (BCSG, *n* = 5), triamcinolone (TG) or their combination group (BCS + TG, *n* = 5). BCSG received a single intra-articular administration of 3 ml of blood cell secretome, TG 0.5 ml of triamcinolone acetonide 40 mg/ml, and BCS + TG received the combined products. The volume to administrate was corrected to 3.5 ml with saline. On days 0, 8, 15, 30, 60, 90, 120, 150, and 180, a copy of the Canine Brief Pain Inventory (divided into pain interference score—PIS and Pain Severity Score—PSS), Liverpool Osteoarthritis in Dogs (LOAD), Hudson Visual Analogue Scale (HVAS), and Canine Orthopedic Index (COI, divided into function, gait, stiffness, and quality of life) was completed. Results were analyzed with the Kruskal–Wallis test and the Kaplan–Meier estimators were conducted and compared with the Log Rank test, *p* < 0.05.

**Results:**

Animals in the sample had a mean age of 9.0 ± 2.9 years and a bodyweight of 28.8 ± 4.1 kg. Hips were classified as moderate (8) and severe (7) osteoarthritis. No differences were found between groups at T0 regarding considered evaluations. Significant differences were observed between groups in pain scores from + 8d- + 150d, with BCS + TG exhibiting better results. The same was observed for HVAS and LOAD, from + 8d- + 120d. Improvements were also observed in several dimensions of the COI. Kaplan–Meier estimators showed that BCS + TG produced longer periods with better results, followed by BCSG and TG.

**Conclusion:**

The intra-articular administration of blood cell secretome improved the clinical signs and scores of several clinical metrology instruments in dogs with hip OA, particularly when combined with triamcinolone. Further studies are required.

## Background

Osteoarthritis (OA) affects the entire joint organ and associated tissues. However, the condition is most commonly associated with the loss and dysfunction of articular cartilage [1]. The disease has a high prevalence, reported to affect 20% of all dogs over one year of age in North America and from 2.5- 6.6% up to 20% of dogs over one year of age in the United Kingdom [2]. This value is expected to rise since the canine population is experiencing a simultaneous rise in life expectancy and prevalence of obesity [3, 4]. Even in working dogs, where obesity is not a common issue, OA still significantly affects the quality of life and performance [5, 6].

There are several therapeutic approaches to the management of OA. Recently, intra-articular treatment modalities have increased interest, particularly regenerative procedures [7–9]. Blood Cell Secretome (BCS) is one of these approaches, and it is based on its content of physiological concentrations of autologous anti-inflammatory mediators and growth factors, including Interleukin 1 receptor antagonist (IL-1Ra), Hepatocyte Growth Factor (HGF), Transforming Growth Factor-β (TGF-β) and Insulin-like growth factor (IGF) [10]. Additionally, factors including lipid mediators, de novo produced exosomes, and mitochondrial signal peptides are present and may support the clinical action [11]. Autologous BCS reduces inflammatory mediators such as IL-1b, NO, and reactive oxygen species. When injected into tendons and OA joints, it can reduce pain and increase weight-bearing in humans [11, 12].

Cytokine-modulating therapies for OA have provided variable clinical results, and there is still a lack of compelling in vivo mechanistic evidence [13]. Therapies focussing on the presence of IL-1ra have been thought to be of great importance since it competitively blocks IL-1 receptors. It is tough that, in cases of degenerative disease, local natural IL-1ra concentrations may be low, making it unable to inhibit the destruction of cartilage, muscle, and other joint structures [14]. There are several reports published on intra-articular IL-1ra, both in animals and humans [15, 16]. Since some questions are recently being raised regarding the effectiveness of targeting IL-1 in the treatment of OA [17, 18]. With that in mind, BCS effects are probably due to the involvement of other factors rather than IL-1ra alone. BCS has been deemed a safe and effective procedure in humans. It was able to reduce pain, with effects lasting up to 2 years, possibly by re-establishing healthy joint homeostasis [11]. Other reports have presented at least one year of improvements, with mean improvements of ± 70% compared to both placebo and hyaluronan in several clinical outcome measures [12, 19]. Currently, there is a lack of information on the intra-articular injection of BCS in dogs.

Different guidelines for the management of human OA provide weak to strong recommendations for using IA corticosteroids [20–24]. IA corticosteroids should be especially considered in patients with moderate to severe pain, showing a poor or no response to oral analgesic medication [25]. Studies in the canine model of OA and patients with natural disease showed that triamcinolone could relieve symptomatic pain and improve mobility [21, 26–28]. It also significantly lead to a smaller osteophyte size and reduced the severity of structural changes of cartilage at the histological level, with no deleterious effects on normal cartilage [29]. In contrast, some reports point to deleterious effects associated with IA corticosteroids, which include the production of a low quantity and high viscosity synovial fluid. However, these findings are often related to multiple injection protocols, specifically of methylprednisolone [30, 31].

Still, and for this reason, a popular approach is the combined administration of corticosteroid and another substance, such as hyaluronan. This combined use of two substances provides a rapid onset of action, obtained from the corticosteroid, associated with a longer effect and decreased possibility of side effects, obtained from hyaluronan [32–34]. Similarly, there is a potential benefit in combining corticosteroids and BCS in a single IA administration. Corticosteroids deliver fast anti-inflammation, immediately calming down the hostile environment and breaking the vicious cycle of ongoing joint degeneration, and BCS offers a long-term build-up of balanced homeostasis. This would enable the joint environment to regenerate while preventing disease progression and protecting from potential detrimental effects of corticosteroids.

Different clinical metrology instruments have been developed to evaluate the various dimensions of OA. Some of the most widely used include the Canine Brief Pain Inventory (CBPI, with two sections, the pain severity score – PSS, and the pain interference score—PIS), and the Liverpool Osteoarthritis in Dogs (LOAD) [35–37]. Other validated instruments include the Canine Orthopaedic Index (COI, divided into four scores: stiffness, gait, function, and quality of life – QOL) and the Hudson Visual Analogue Scale (HVAS) [38–40].

This study aimed to evaluate the intra-articular treatment with the combination of BCS and triamcinolone in dogs with bilateral hip OA. We hypothesized that the combined use would better alleviate joint pain while improving other OA-related clinical signs than their isolated use.

## Results

The sample included 15 active police working dogs, with a mean age of 9.0 ± 2.9 years and bodyweight of 28.8 ± 4.1 kg, representing both sexes (8 males and 7 females). Four dog breeds were represented, similarly distributed between the groups: German Shepherd Dogs (*n* = 11), Labrador Retriever (*n* = 2), Belgian Malinois Shepherd Dogs (*n*= 1), and Dutch Shepherd Dog (*n* = 1). Eight hips were classified as having moderate OA and 7 as severe. All patients were followed up to the 180 days evaluation time points. Although the exact volume of BCS produced was not measured, an excess of 6 ml per device was obtained, as a single device rendered enough BCS to treat both hips. The BCS produced with the second device was frozen for future treatment for each dog.

**Fig. 1:**
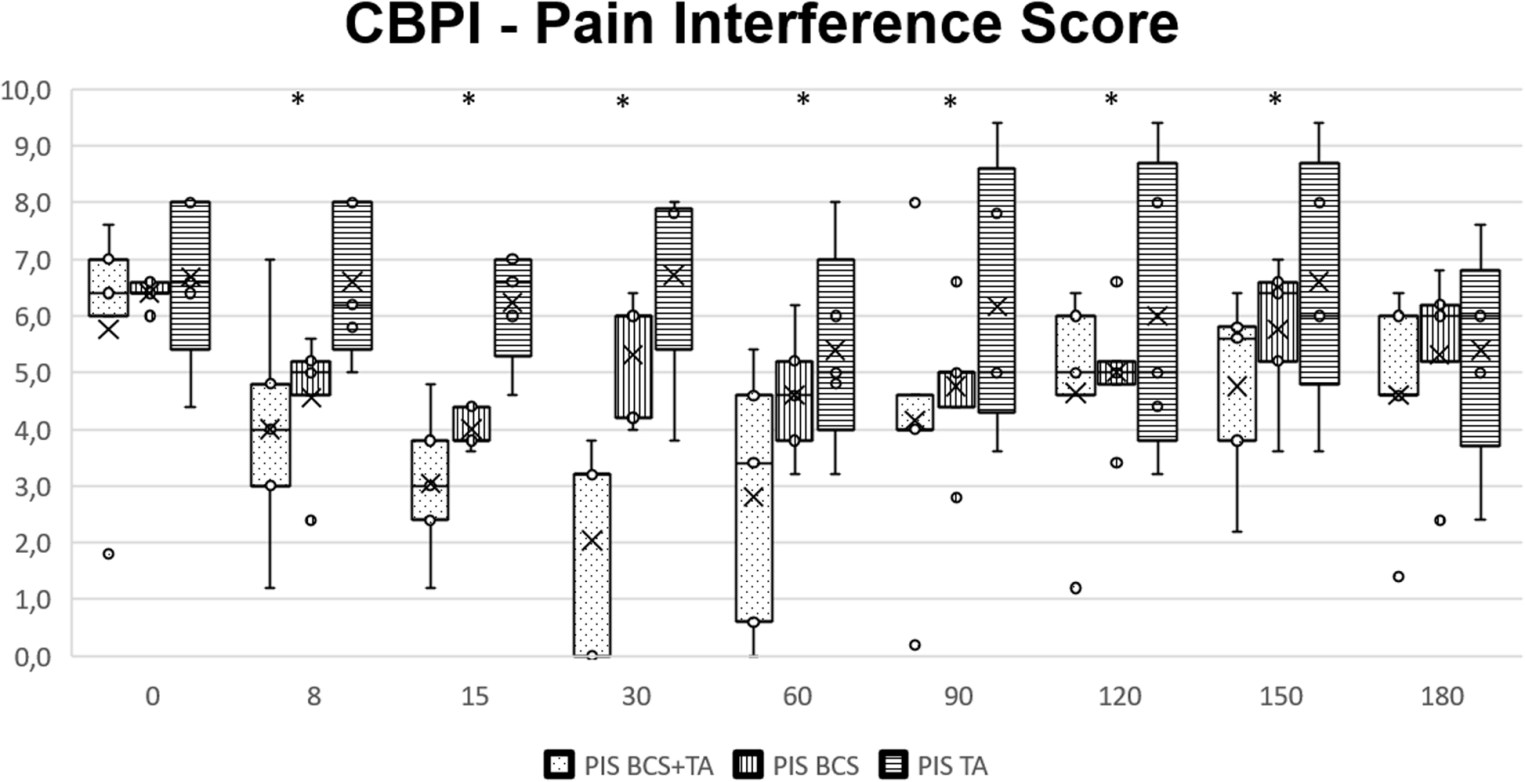
Overall evolution of Pain Interference Score in the three groups. Box plots represent the median, 25th and 75th percentiles, and whiskers represent 10th and 90th percentiles. * indicates significant differences between groups

No additional treatment or medications were administered. The evolution of CMI scores for all groups is presented in Table 1. Significant differences were observed in both scores of the CBPI from the 8 days after treatment, up to the 150-day evaluation, with BCS + TG showing lower scores than the remaining groups. The evolution of PIS is shown in Fig. 1. Similar results were observed with the LOAD and HVAS, with improvements lasting up to the 120-day evaluation. With the different dimensions of the COI, improvements in Stiffness, Function, and Gait were observed between the 15 days and 90 days evaluations.

**Table 1:** Median values and interquartile range (IQR) of the clinical metrology instruments evaluated throughout the study

Clinical Metrology Instrument		Group	T0		*p*	+ 8d		*p*	+ 15d		*p*	+ 30d		*p*	+ 60d		*p*	+ 90d		*p*	+ 120d		*p*	+ 150d		*p*	+ 180d		*p*
			**Med**	**IQR**		**Med**	**IQR**		**Med**	**IQR**		**Med**	**IQR**		**Med**	**IQR**		**Med**	**IQR**		**Med**	**IQR**		**Med**	**IQR**		**Med**	**IQR**	
**CBPI**	**PIS**	BCS + TG	6.4	1,0	0.17	4,0	1.8	0.04*	3,0	1.4	0.01*	3.2	3.2	< 0.01*	3.4	4,0	0.02*	3,0	0.6	0.03*	3,0	1.4	0.04*	4,0	2,0	0.03*	4.6	1.4	0.64
		BCSG	5.2	0.2		5,0	0.6		3.8	0.6		6,0	1,0		4.6	1.4		5,0	0.6		4,0	0.4		6.4	1.4		6,0	1,0	
		TG	6.6	1.6		6.2	2.2		6.6	1,0		7,0	0.8		5,0	1.2		5,0	2.8		5,0	3.6		6.4	2,0		6,0	1,0	
	**PSS**	BCS + TG	5.3	2.3	0.17	4.25	2.25	0.03*	2.5	2.3	0.02*	3,0	1.75	0.02*	4,0	3.25	0.02*	4,0	1.25	0.03*	4,0	1.8	0.02*	4.3	2,0	0.04*	5,0	1,0	0.92
		BCSG	4.5	1.5		4,0	1.75		3.8	1,0		4,0	0.75		4.8	0,0		4.8	0.5		5,0	0.3		5.3	0.5		5,0	0.5	
		TG	6,0	1.5		5.75	0.5		6,0	0.8		7,0	0.5		5.8	1.25		5.3	2.5		5,0	2.8		6,0	2.75		4.8	1.8	
**HVAS**		BCS + TG	5.2	2.4	0.88	3.2	0.1	0.01*	3.7	0.5	0.03*	3.3	0.8	0.02*	3.3	0.9	0.03*	4,0	0.5	0.04*	4.5	2.5	0.88	4.5	2.1	0.69	5.2	2,0	0.99
		BCSG	4.2	0.5		4.6	1,0		4.5	1,0		4.4	0.6		4.2	0.6		4.9	0.6		4.2	0.5		4.6	1.6		4.6	1,0	
		TG	4.4	1.5		4.5	0.1		4.5	1.2		4.8	1.3		4.5	2,0		5.2	1.5		4.5	0.3		4.5	1.3		4.8	0.8	
**LOAD**		BCS + TG	28,0	16,0	0.31	15,0	4,0	0.04*	17,0	8,0	0.04*	22,0	10,0	0.03*	23,0	2,0	0.03*	25,0	11,0	0.04*	22,0	12,0	0.04*	27,0	14,0	0.88	29,0	11,0	0.85
		BCSG	26,0	2,0		22,0	3,0		21,0	2,0		21,0	3,0		25,0	0,0		25,0	2,0		25,0	3,0		23,0	4,0		23,0	3,0	
		TG	27,0	8,0		26,0	4,0		25,0	4,0		25,0	1,0		28,0	6,0		32,0	3,0		35,0	6,0		23,0	2,0		23,0	4,0	
**COI**	**Stiffness**	BCS + TG	8,0	2,0	0.18	5,0	2,0	0.57	4,0	3,0	0.03*	4,0	4,0	0.03*	5,0	0,0	0.02*	6,0	4,0	0.04*	8,0	4,0	0.88	8,0	4,0	0.27	8,0	4,0	0.87
		BCSG	6,0	2,0		6,0	0,0		4,0	3,0		4,0	1,0		6,0	2,0		7,0	0,0		7,0	3,0		7,0	2,0		7,0	3,0	
		TG	11,0	3,0		8,0	8,0		8,0	2,0		8,0	2,0		8,0	0,0		8,0	6,0		8,0	6,0		8,0	2,0		8,0	1,0	
	**Function**	BCS + TG	9,0	7,0	0.3	7,0	2,0	0.75	5,0	5,0	0.02*	5,0	4,0	0.04*	6,0	0,0	0.03*	6,0	4,0	0.04*	8,0	5,0	0.83	8,0	6,0	0.58	10,0	7,0	0.82
		BCSG	7,0	2,0		7,0	1,0		7,0	2,0		7,0	0,0		7,0	1,0		8,0	0,0		8,0	1,0		7,0	2,0		8,0	1,0	
		TG	11,0	5,0		8,0	7,0		8,0	3,0		8,0	4,0		8,0	0,0		8,0	6,0		8,0	7,0		8,0	4,0		8,0	0,0	
	**Gait**	BCS + TG	12,0	5,0	0.29	10,0	4,0	0.33	8,0	7,0	0.02*	8,0	5,0	0.02*	9,0	1,0	0.04*	9,0	3,0	0.04*	10,0	7,0	0.03*	10,0	6,0	0.45	11,0	4,0	0.72
		BCSG	10,0	3,0		10,0	1,0		9,0	1,0		9,0	1,0		10,0	0,0		10,0	1,0		11,0	1,0		10,0	2,0		11,0	1,0	
		TG	13,0	4,0		13,0	3,0		13,0	3,0		13,0	2,0		13,0	2,0		14,0	7,0		14,0	6,0		13,0	6,0		13,0	4,0	
	**QOL**	BCS + TG	6,0	1,0	0.14	5,0	1,0	0.29	5,0	3,0	0.26	6,0	1,0	0.15	6,0	1,0	0.03*	6,0	2,0	0.04*	7,0	2,0	0.03*	8,0	1,0	0.49	7,0	1,0	0.13
		BCSG	6,0	0,0		6,0	1,0		6,0	1,0		5,0	1,0		6,0	0,0		6,0	0,0		6,0	0,0		6,0	1,0		6,0	1,0	
		TG	9,0	2,0		7,0	2,0		7,0	0,0		9,0	2,0		8,0	2,0		9,0	4,0		9,0	3,0		7,0	2,0		7,0	1,0	
	**Overall**	BCS + TG	35,0	15,0	0.13	27,0	8,0	0.01*	22,0	19,0	0.03*	23,0	14,0	0.02*	26,0	2,0	0.04*	27,0	13,0	0.01*	33,0	16,0	0.04*	34,0	15,0	0.04*	36,0	16,0	0.6
		BCSG	29,0	9,0		29,0	6,0		26,0	2,0		25,0	1,0		29,0	6,0		31,0	3,0		32,0	4,0		30,0	3,0		32,0	3,0	
		TG	44,0	13,0		36,0	20,0		36,0	11,0		38,0	6,0		37,0	4,0		39,0	25,0		39,0	23,0		36,0	15,0		36,0	4,0	

*CBPI* Canine Brief Pain Inventory, *COI* Canine Orthopedic Index, *HVAS* Hudson Visual Analogue Scale, *LOAD* Liverpool Osteoarthritis in Dogs, *PIS* Pain Interference Score, *PSS* Pain Severity Score, *QOL* Quality of Life. *P* values for the comparison between both groups at each follow-up moment are presented

^*^ indicates significance

**Fig. 2:**
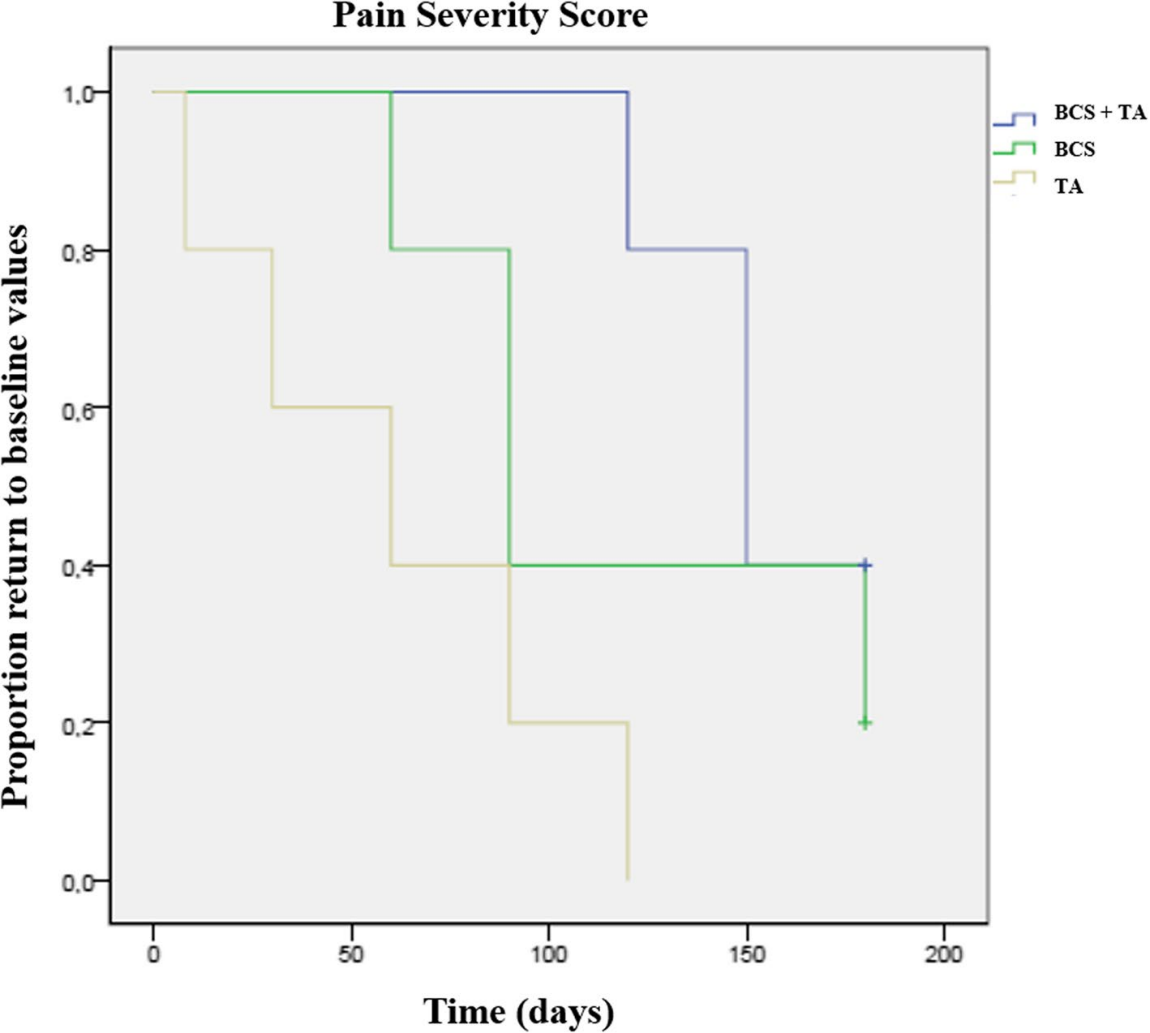
Kaplan–Meier curve demonstrating a significant difference between the three treatment groups, in time for Pain Severity Score to drop below a one point improvement (*p* < 0.01)

**Fig. 3:**
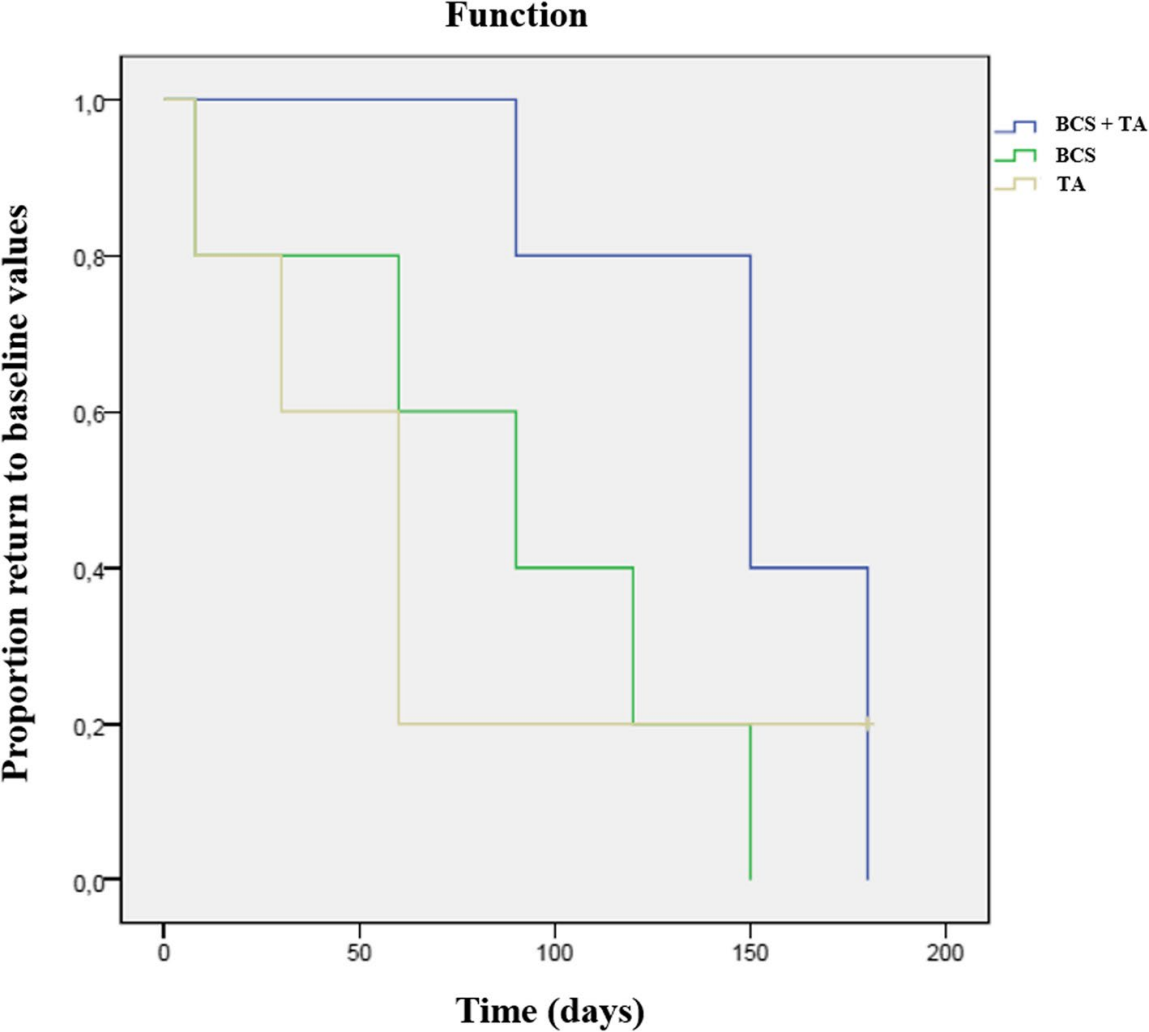
Kaplan–Meier curve demonstrating a significant difference between the three treatment groups, in time for the Function dimension of the Canine Orthopedic Index to return to baseline values (*p* = 0.03)

Results of the Kaplan–Meier estimators with each evaluation method are presented in Table 2, and Figs. 2 and 3 present Kaplan Meier plots for PIS and COI's Function dimension. BCS + TG showed more extended periods with better results, with patients taking longer to return to baseline values and scores, followed by BCSG and TG. No side effects were recorded in either group.

**Table 2:** Time to return to baseline values for the clinical metrology instruments considered, calculated with Kaplan–Meier estimators and compared with the Log Rank test

			**Group**											
**Clinical Metroly Instrument**		**Log Rank test**	**BCS + TG**				**BCSG**				**TG**			
			**mean**	**SD**	**95% CI**		**mean**	**SD**	**95% CI**		**mean**	**SD**	**95% CI**	
CBPI	PSS	0.02*	156.0	10.4	136.3	175.7	120.0	25.9	69.2	170.8	61.6	20.1	22.2	101.0
	PIS	< 0.01*	168.0	7.6	153.1	182.9	102.0	7.3	87.6	116.4	72.0	22.5	27.9	116.0
HVAS		0.02*	162.0	13.1	136.2	187.8	123.0	32.6	59.0	186.9	73.6	18.9	36.5	110.7
LOAD		0.86	115.6	33.1	50.7	180.5	121.6	28.1	66.5	176.6	97.6	31.3	36.3	158.9
COI	Stiffness	0.04*	120.0	24.8	48.9	146.3	97.6	14.7	91.2	148.8	70.6	27.9	15.9	125.3
	Function	0.03*	150.0	16.4	117.8	182.2	85.6	24.5	37.5	133.7	67.6	26.6	15.4	119.8
	Gait	0.03*	180	0.0	180.0	180.0	132.0	15.3	102.0	161.9	73.6	26.8	21.0	126.2
	QOL	0.04*	132.0	7.3	117.6	146.4	90.0	16.4	57.8	112.2	79.6	28.1	24.6	134.6
	Overall	0.02*	174.0	6.6	161.1	186.9	132.0	18.2	96.3	167.7	73.6	26.8	21.0	126.2

*CBPI* Canine Brief Pain Inventory, *COI* Canine Orthopedic Index, *HVAS* Hudson Visual Analogue Scale, *LOAD* Liverpool Osteoarthritis in Dogs, *PIS* Pain Interference Score, *PSS* Pain Severity Score, *QOL* Quality of Life. *P* values for the comparison between both groups at each follow-up moment are presented

^*^ indicates significance

## Discussion

Osteoarthritis still presents a significant welfare problem for many dogs, with limited therapeutic options other than palliative pain control [41, 42]. Our results show that the IA administration of BCS, particularly in combination with triamcinolone, can reduce pain levels and improve scores of several clinical metrology instruments in dogs with hip OA.

Currently, there are no controlled study reports on the efficacy of BCS, alone or in combination with corticosteroids, in managing canine OA. Other Autologous Blood Products share some similarities with BCS. Autologous Blood Products are based on their content of supra physiological concentrations of autologous anti-inflammatory mediators and growth factors or cells containing these proteins, leading to a reduction in inflammatory [43–46]. Autologous Blood Products can reduce pain and lameness scores and increase weight bearing when injected into OA joints [47–49], superior to both placebo and hyaluronan in several clinical outcome measures [12, 19]. In dogs, studies have shown that Autologous Blood Products can produce improvements up to 12 weeks, with increased activity levels, decreased lameness, and pain [48, 50]. Our results showed that BCS improved the scores of several clinical metrology instruments, particularly pain scores. In particular, the combination of BCS with triamcinolone produced significantly lower pain scores from the first follow-up evaluation. This early improvement is probably due to the effect of triamcinolone. Many scores in BCS + TG were significantly better up to the + 120d follow-up and, in the case of PIS and PSS, up to + 150d evaluation. Similar results were observed with the Kaplan Meier test, with BCS + TG scores taking longer to return to baseline values, followed by BCS and TG. Although a small number of animals were enrolled in this first study, these results are interesting and positive. Particularly, considering that a large proportion of animals in this sample had severe OA, in contrast with previous studies, in which intra-articular modalities were evaluated [28, 51]. In addition, all animals were treated in August, and for that reason, the + 120d to + 180d evaluations were conducted during the colder months of the year, with a toll on OA patients. Still, we saw an improvement, particularly in pain scores, during this period. The results observed in TG are in line with previously reported effects of triamcinolone [7, 27]

Clinical evidence shows that autologous conditioned serum can improve joint homeostasis, as can PRP [11]. Still, a growing body of evidence indicates that other peripheral blood cells can release biologically active paracrine factors that can produce regenerative effects [52], specifically in joint cartilage [53, 54]. In a rat model, the addition of conditioned medium secretome from mesenchymal stem cells leads to lower levels of inflammatory factors and better results than a control group [55]. Although many of the animals included in the sample already had severe OA and still showed improvements, there is a case to be made for earlier use of BCS, as its effects are produced through the interaction with the different types of joint cells and tissues, so they may need to be present in enough number for a more significative response to be observed and to preserve healthy tissues [28].

Documented side effects of intra-articular administrations include local pain and local inflammation, usually self-limiting and taking 2–10 days to resolve spontaneously [28]. Some patients showed complaints following the administration in all groups but resolved spontaneously and did not impact the first follow-up evaluation. The study presents some limitations, namely the size of the sample. This first report served as a proof of concept, and future studies should enroll more animals. Although all instruments used have been validated, and several were used, future studies should include an objective evaluation, such as Force Plate Gait Analysis or Stance Analysis. Analysis of synovial fluid inflammatory markers and molecular and cellular characterization of BCS is also of interest and should be considered in future research.

## Conclusions

The intra-articular administration of blood cell secretome improved the clinical signs and scores of several clinical metrology instruments in dogs with moderate to severe hip OA. In particular, its combined use with triamcinolone produced an earlier reduction in pain scores and a long-lasting effect. Further studies are required.

## Methods

This preliminary study selected fifteen patients, constituting a convenience sample, based on history, physical, orthopedic, neurological, and radiographic examinations compatible with bilateral hip OA [5]. Additional inclusion criteria comprise age over two years and a body weight over 15 kg. Patients should not have received any medication or nutritional supplement for over six weeks [7]. Cases of documented or suspected orthopedic or neurological disease, or any other concomitant disease, were excluded. After selection, patients were randomly distributed between groups with the statistical analysis software to a BCS group (BCSG, *n* = 5), triamcinolone group (TG, *n* = 5), and a Blood Cell Secretome + triamcinolone group (BCS + TG, *n* = 5). In all patients, both hips were treated.

Patients in BCSG received a single IA administration of 3 ml of Blood Cell Secretome per hip joint, prepared with a commercial kit (Orthogen® Device, Orthogen AG, Düsseldorf, Germany), following the manufacturer's guidelines. Briefly, 15 ml of whole blood was collected per device, from the jugular vein in the morning, with the patient fasted. The blood was collected directly to the device, and 2 devices were prepared (since the manufacturer indicates that 5 ml of BCS can be obtained from each device, and a total of 6 ml are required to treat both hips). After blood collection, the devices were immediately placed in the appropriate rack and incubated for 4.5 h at 37 °C (MF-6 W incubator, HCP-Technology, Nortrup, Germany). After incubation, the devices were centrifuged for 3 min at 1500 g (M-Universal, MPW, Warsaw, Poland). Finally, the vial containing the sterile filtered Blood Cell Secretome was collected. Patients in TG received an IA administration of 20 mg in a volume of 0.5 ml of triamcinolone acetonide (Triam Lichtenstein, Zentiva, Germany) per hip joint. Patients in BCS + TG received an IA combined administration of the two products (3 ml of BCS and 0.5 ml of triamcinolone acetonide, 3.5 ml in total volume). To provide an equal volume per hip joint in all groups, the total volume in BCS and TG was corrected with saline to obtain a final volume of 3.5 ml. The syringe was covered, to mask the administered treatment’s appearance.

The patients were placed under light sedation for the radiographic examination and IA administrations, induced with a combination of medetomidine (0.01 mg/kg) and butorphanol (0.1 mg/kg), given intravenously [56]. Joints were classified according to the Orthopedic Foundation for Animals hip grading scheme [57], based on a standard ventrodorsal extended legs projection. These procedures were conducted by the same researcher, blinded to the animal's assigned group. The syringes containing the different products were prepared by a different researcher and covered to look the same. The procedure for intra-articular administration has been previously described [58]. Patients were placed in lateral recumbency, with the limb of the joint being accessed uppermost. A window of 4 × 4 cm in the area surrounding the greater trochanter was clipped and aseptically prepared. With the limb placed in a neutral position, parallel to the table, a 2,5" 21-gauge needle was introduced just dorsal to the greater trochanter, perpendicular to the long axis of the limb, until the joint was reached. Confirmation of correct needle placement is obtained through the collection of synovial fluid. After removing as much synovial fluid as possible, the treatment was administered. After treatment, the animals rested for three consecutive days and resumed their regular activity over 5 days. The need for rescue analgesia was recorded.

On treatment day, 8, 15, 30, 60, 90, 120, 150, and 180 days post-treatment, the dog's handler completed a digital copy of the CBPI, LOAD, HVAS, and COI after receiving the published instructions for each of them. They are to be completed sequentially by the same person in each follow-up assessment, without knowing their previous answers, and blinded to treatment in a quiet room with as much time as needed to answer all items. Since an improvement with HVAS consists of an increased score, while the opposite occurs in the remaining CMIs, HVAS scores were inverted by subtracting the result from 10 (the higher possible range score) to facilitate interpretation of the results.

### Statistical analysis

Normality was assessed with a Shapiro–Wilk test. In each evaluation moment, groups were compared using a Kruskal – Wallis test. The Kaplan–Meier was performed to generate time to event curves, and event probability and results were compared with the Log Rank test. With the CBPI, a specific measure of success has been defined, set as a reduction of ≥ 1 in PSS and ≥ 2 in PIS [59]. For these scores, the Kaplan–Meier test was used to evaluate the time for the score to drop below these improvement levels. No specific measure of success is published for the LOAD, HVAS, and COI. For that reason, we considered the outcome a return to, or drop below, the initial values of CMI scores at the 180-day evaluation. The rationale for this selection was based on the fact that it motivated the need for medical assistance [8, 28]. Patients with scores above baseline values at the final evaluation moment were censored. All results were analyzed with commercially available software (IBM SPSS Statistics version 20), and a significance level of *p* < 0.05 was set.

## Data Availability

The datasets used and/or analyzed during the current study are not readily available because the data used in this study is the property of the Guarda Nacional Republicana, a governmental police force from Portugal, and, by law, confidential. Access to the datasets is available from the corresponding author on reasonable request.

## Abbreviations

CBPI: Canine Brief Pain Inventory
COI: Canine Orthopedic Index
HVAS: Hudson Visual Analogue Scale
LOAD: Liverpool Osteoarthritis in Dogs
OA: Osteoarthritis
PIS: Pain Interference Score
PSS: Pain Severity Score
QOL: Quality of Life
